# Keystone Predation: What Is It, and Is It Supported by Empirical Evidence?

**DOI:** 10.1002/ece3.72488

**Published:** 2025-12-11

**Authors:** Anthony J. Gillis, Mads S. Thomsen, Jonathan D. Tonkin

**Affiliations:** ^1^ School of Biological Sciences University of Canterbury Christchurch New Zealand; ^2^ UWA Oceans Institute & School of Biological Sciences University of Western Australia Crawley Western Australia Australia; ^3^ Department of Ecoscience Arhaus University Roskilde Denmark; ^4^ Te Pūnaha Matatini Centre of Research Excellence University of Canterbury Christchurch New Zealand

**Keywords:** biodiversity, competition, ecological interactions, ecological mechanism, indirect effects, keystone predation, predation

## Abstract

Keystone predation is the process whereby a predator indirectly facilitates weak competitors by preferentially consuming strong competitors, often affecting local diversity. The keystone predation process is therefore different from keystone species, which refers to any species with a large and disproportionately large effect relative to its abundance. Keystone predation is widely cited as a fundamental ecological process in textbooks and scientific literature, but its empirical evidence has not been reviewed. We addressed this research gap by reviewing papers that explicitly (a) tested for keystone predation with experiments or models, and/or (b) mentioned ‘keystone predation’ in their main text, to assess its prevalence and identify potential context dependencies. We found that almost 2000 publications mentioned keystone predation in their texts, but only 73 papers tested or discussed the process in detail. Most of the 73 studies were from North America (87.4%), temperate (59.4%) and aquatic habitats (48% freshwater, 44% marine). Just 25 publications manipulated predator abundances to explicitly test for keystone predation, of which 10, 7 and 8 publications reported consistent support (mobile invertebrates preying on sessile competitors), context‐dependent support (effects varied, e.g., with temperature or nutrients) or no support (mobile competitors), respectively. The few studies that tested keystone predation make it difficult to evaluate its prevalence, its relative importance, or whether general rules exist to predict its strength. Still, the few published studies suggest that environment, species traits and dispersal can modify keystone predation. We also recognise that more papers may have tested for keystone predation, but lacked this terminology. These papers should be identified in future meta‐analyses by combining wider search terms and expert knowledge. We recommend that researchers use the precise keystone predation terminology to experimentally test this process on different consumer–prey communities and under contrasting environmental conditions, to better understand its prevalence and importance in ecology.

## Graphical abstract

Keystone predation has been widely cited as a key ecological mechanism controlling community structures, but is supported by limited theoretical and experimental studies. Reviewing these studies showed keystone predation has been documented across realms, appears to be most prevalent in sessile and dispersal‐limited communities and can be modified by environmental conditions, species traits and dispersal. Therefore, more research that explicitly tests for keystone predation on different predator–prey communities and under contrasting environmental conditions is needed to better understand its prevalence and importance in ecology.

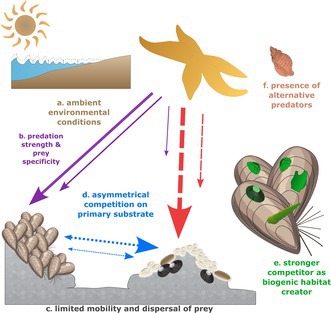

## 1 Introduction

In 1966, Robert Paine published a seminal paper demonstrating that the sea star *Pisaster ochraceus* preferentially consumed mussels in rocky intertidal communities, opening space for a diverse set of weaker sessile competitors (Paine 1966). This process, later coined keystone predation, occurs when a predator indirectly facilitates a weaker competitor by reducing a dominant competitor's abundance via preferential feeding, often affecting local species diversity (Box 1; Paine 1966, Paine 1969). Paine's experiment famously showed that removing *P. ochraceous* from rocky intertidal communities reduced predation on the mussel *Mytilius californicus*, which subsequently monopolised space and outcompeted weaker sessile competitors like barnacles, chitons and limpets, reducing diversity of primary space holders from 18 to 9 species (Lafferty and Suchanek 2016; Paine 1966, 1969). Keystone predation has since been broadened to include all processes whereby consumers (typically predators like sea stars, but can also be herbivores; see Poelman and Kessler 2016) preferentially consume a strong competitor to indirectly facilitate an inferior competitor (Lafferty and Suchanek 2016; Menge 1995; Wootton 1994), thereby differing from other indirect interactions (Box 1). The keystone predation process thereby also differs from the derived keystone species concept, which defines species (i.e., objects, not processes) whose effects are large and disproportionately large relative to their abundance (sensu Power et al. 1996, Box 1). The keystone species concept has, in contrast to the keystone predation process, already been reviewed in detail (Cottee‐Jones and Whittaker 2012; Davic 2003; Mills and Doak 1993; Mouquet et al. 2013; Paine 1995; Power et al. 1996; Power and Mills 1995; Valls et al. 2015) and is not addressed here. Paine's seminal papers on keystone predation are today considered among the most influential ecological research (Lafferty and Suchanek 2016). Over the past 50 years, keystone predation has been widely cited as a key ecological process (Lafferty and Suchanek 2016), has been taught in many ecological textbooks (Krebs 2009; Mittelbach and McGill 2019; Smith 2015), and has been discussed in reviews of indirect species effects (Menge 1995; Paine 1995; Wootton 1994). However, the broad applicability of keystone predation is still uncertain, as it often depends on context (Chamberlain et al. 2014; Harley 2011; Sanford 1999) and is prone to conceptual misinterpretation (see discussion and Box 1; Lafferty and Suchanek 2016; Power et al. 1996). Considering the presumed broad importance of keystone predation, its empirical underpinnings should be confirmed to ensure scientific understanding is based on quantitative evidence.

BOX 1 **Defining ecologically important species and processes.**

**FIGURE 1:**
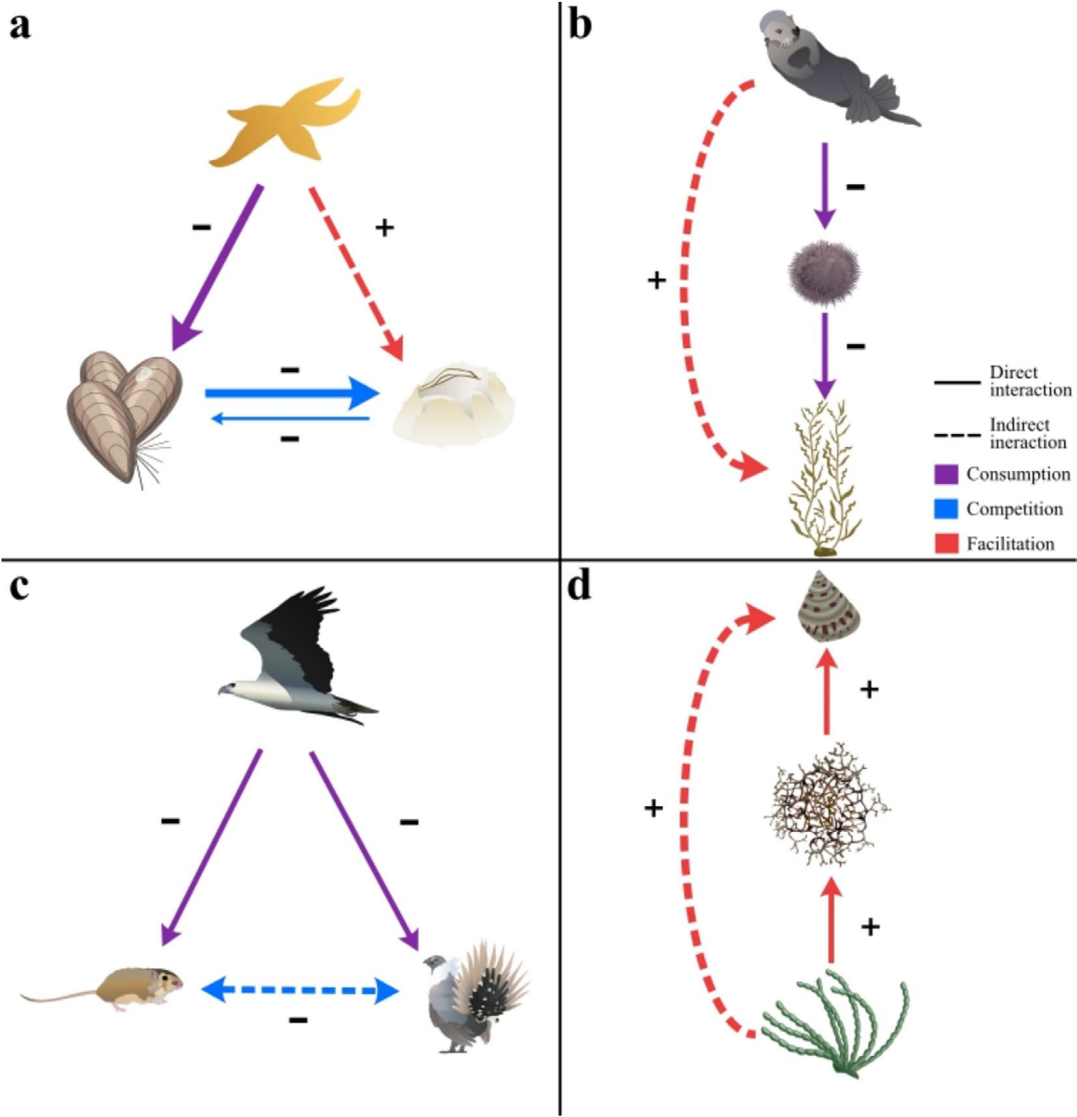
(Box 1) Conceptual diagrams comparing common forms of indirect species interactions, including (a) keystone predation (sea star–mussel–barnacles; Paine 1966), (b) trophic cascade (otter–urchin–kelp; Estes et al. 1998), (c) apparent competition (hen harrier‐red grouse‐vole; Barraquand et al. 2015), and (d) facilitation cascade (rockweed–epiphyte–invertebrate; Thomsen et al. 2016). Solid lines = direct interactions and dashed lines = indirect interactions. Colours of the lines represent consumption (purple), competition (blue), and facilitation (red) interaction types. The plus (+) and minus (−) symbols represent inhibition and facilitation. Arrows depict the direction of impact between levels, rather than the flow of energy. Species graphics are from the Integration and Application Network (ian.umces.edu/media‐library). They are licenced under Attribution‐ShareAlike 4.0 International (CC BY‐SA 4.0).

Robert Paine's sea star removal experiment (Paine 1966) has served as a critical inflexion point in ecology, leading to debates on how ecologists should classify and codify the crucial elements underpinning an ecosystem's diversity (Figure 1). It is important to emphasise here that species identities and processes are distinct, such as between keystone predator, the identity and keystone predation, the process. We define some key terms below, including whether they are objects or processes.

- *Keystone species*: A species (i.e., object) whose effect is large and disproportionately large relative to its abundance (Paine 1969; Power et al. 1996). Keystone species may be found across different taxa (vertebrates, invertebrates or plants), feeding methods (predator, herbivore, parasite) and can occupy different trophic positions (producer, consumer). They may also be found in various roles and functions in the community, such as habitat formers or consumers. A keystone species is typically considered a binary distinction (e.g., to be a keystone vs. not), but some researchers argue this distinction should be made using a quantifiable continuous trait, such as ‘keystoneness’ (Cagua et al. 2019; Jordán et al. 2006; Power et al. 1996).
- *Foundation species*: A species (i.e., object) that creates or maintains habitat for other species, modulates ecosystem processes and plays a strong role in structuring the community that is proportionate to its abundance (Ellison 2019; Lamy et al. 2020; Thomsen et al. 2018). A foundation species is largely synonymous with Power et al. (1996) ‘dominant’ species.
- *Keystone predation*: The process whereby a consumer preferentially consumes a strong intermediary competitor species, indirectly facilitating a weak basal competitor (often affecting biodiversity, Figure 1a; Menge et al. 2021; Paine 1966, 1969).
- *Trophic cascade* (sometimes referred to as a consumption cascade, Thomsen et al. 2010): the process whereby a predator indirectly facilitates a basal species by consuming an intermediate consumer (often affecting community structure and biodiversity, Figure 1b; Borer et al. 2005; Paine 1980; Shurin et al. 2002).
- *Apparent competition*: The process whereby a generalist species benefits from (i.e., increasing its abundance) consuming two basal species, that thereby ‘appear’ to compete with each other (instead, each of the two prey species support higher survival, growth and fecundity of the consumer, Figure 1c; Holt 1977; Holt et al. 1994; Holt and Bonsall 2017).
- *Facilitation cascade*: The process whereby a basal species facilitates an intermediary species, which in turn facilitates tertiary species, such that the basal species facilitates the tertiary (Figure 1d; Altieri et al. 2007; Thomsen et al. 2010, 2018).

We are not aware of any reviews or meta‐analyses that have collated and analysed scientific findings on keystone predation, in contrast to analyses of other indirect effects, such as trophic cascades, apparent competition and facilitation cascades (Brett and Goldman 1996; Holt and Bonsall 2017; Shurin et al. 2002; Thomsen et al. 2010, 2018). Here, we target this research gap by reviewing studies that explicitly (a) mention and (b) test the keystone predation process (Box 1). From these studies, we determined the global distribution of keystone predation studies, potential context dependencies that modulate its strength, and identified the realms, habitats and community characteristics in which it has been studied. We also discuss confusion around definitions for keystone predation as a process (reviewed here) versus the keystone species concept, and finish by providing recommendations for future studies to better understand keystone predation.

## 2 Methods

We conducted several literature searches using Google Scholar and Scopus to identify the prevalence of keystone predation in the scientific literature. First, we examined how often keystone predation is mentioned compared to other well‐described indirect ecological interactions by searching for *“keystone predation”*, *“trophic cascade”*, *“facilitation cascade”, “apparent competition”*, *“exploitation competition”*, or *“exploitative competition”* in Google Scholar on November 5th, 2023. We did not include a “keystone predat*” search term because this also returns publications about the “keystone predator” concept, which we intentionally avoid. Second, we narrowed the search to focus on peer‐reviewed publications by searching for *“keystone predation”* in the *full‐text* search field in Scopus on November 13, 2023. The papers identified in this expansive full‐text search could potentially only describe ‘keystone predation’ superficially. We therefore randomly selected 100 of these publications and recorded which manuscript section(s) ‘keystone predation’ was mentioned (abstract, keywords, introduction, methods, results, discussion or references). From this search, we tallied the proportion of papers where keystone predation was discussed in the text vs. simply identified because a cited paper included ‘keystone predation’ in its title. Third, to identify the publications where keystone predation was the primary research goal, we searched for ‘keystone predation’ in the *title, abstracts, and keywords* fields in Scopus on November 14, 2023. We then searched for journals, books and reports that use “ecology” OR “ecological” between the years 1993 and 2023 to standardise temporal trends in our second and third searches. These publications were removed from the *full document* returns to avoid double‐counting. Finally, because Scopus only identified publications from 1993 onwards, we added another Google Scholar search on December 19, 2023, searching for ‘keystone predation’ literature between 1966 and 1993.

To identify research gaps and better understand the prevalence and context dependencies in keystone predation, metadata were extracted from each publication returned from the third (Scopus) and final (Google Scholar) searches. Specifically, we extracted information related to (a) publication year, (b) experimentation type (mensurative, manipulative, mathematical model, nonexperimental/review), (c) experimental location (field, laboratory, mesocosm), (d) realm (marine, freshwater terrestrial), (e) predator manipulation (yes, no), (f) predator/competitor taxa (vertebrate, invertebrate, plants, bacteria, protists), (g) predator/competitor mobility (sessile, mobile) and (h) available latitude/longitude (for field studies and collections areas for taxa used in laboratory or mesocosm experiments). Data across the terrestrial, freshwater and marine realms were also reclassified in tropical (0–23.5), subtropical (23.5–40), temperate (40–60) and polar (60–90) climatic zones. Note that because we identified surprisingly few empirical tests for keystone predation (see results sections), data extraction for a global meta‐analysis of interaction strengths was not feasible. Instead, we used simple vote counting to quantify the distribution of experiments in each metadata category. Furthermore, *χ* ^2^ tests were used to examine whether individual categories were studied evenly (null hypothesis). Finally, we summarised whether the author(s) of predator manipulation experiments concluded that they found evidence for keystone predation.

## 3 Results

**FIGURE 2:**
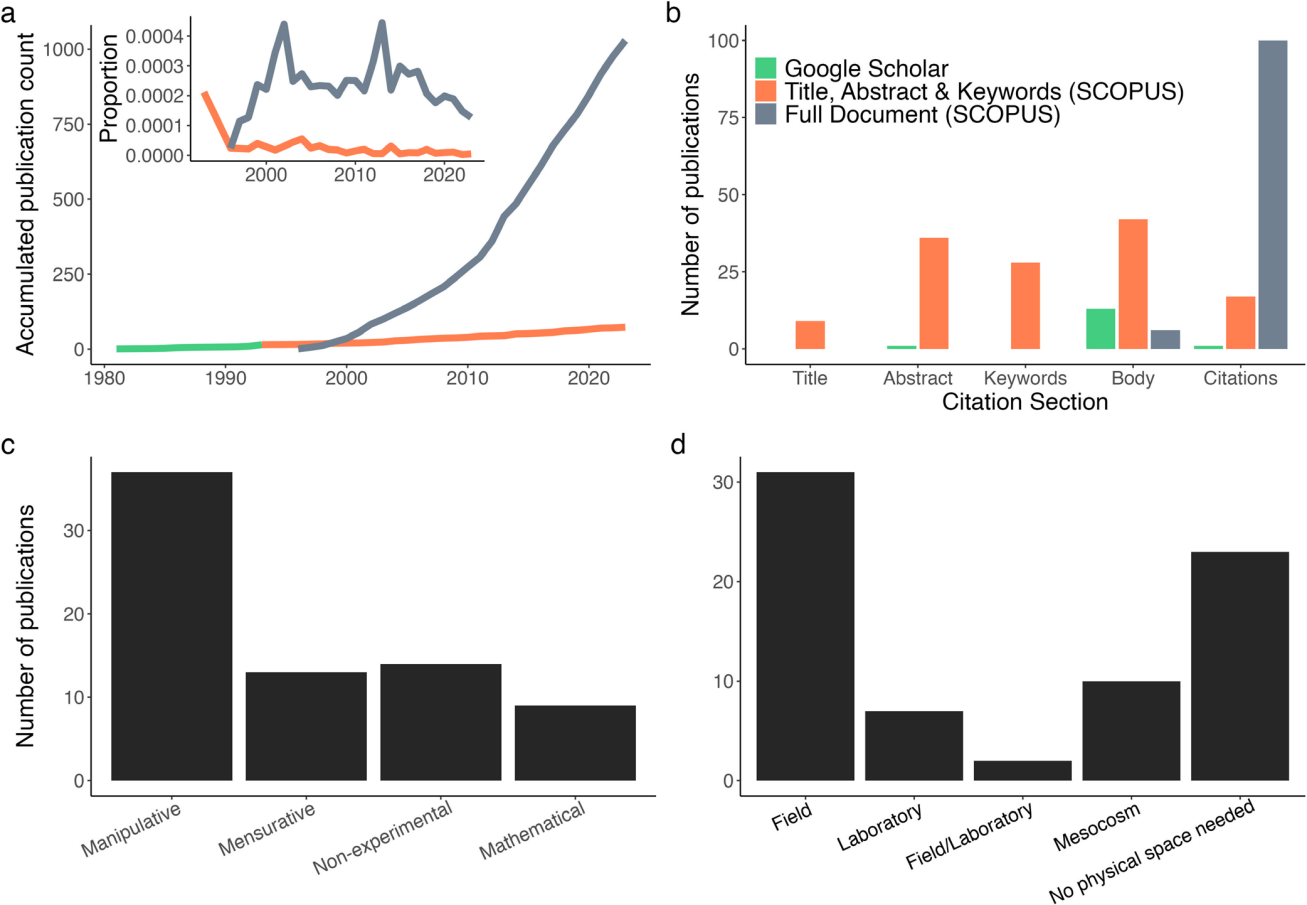
Overview of keystone predation in the literature. (a) Keystone predation publications accumulated through time in different databases and their proportion compared to all ecological literature (inset), (b) analysis of sections where keystone predation was only briefly mentioned (grey = Scopus 1993–2023, *n* = 100) or was explicitly tested and/or discussed in detail (green = Google Scholar 1981–1993, orange = Scopus 1993–2023, *n* = 73). (c) The number of publications that explicitly tested or mentioned keystone predation, categorised by experimental approach and (d) venue of data collection.

The first broad Google Scholar search returned 1910 publications for ‘keystone predation’, which was less than trophic cascades (25,100), apparent competition (15,900) and exploitative/exploitation competition (14,300) but more than facilitation cascades (543). By comparison, the second Scopus search (in full document) returned 852 publications, the third Scopus search (in *title, abstract and keywords*) 58 publications, and the last broad Google Scholar search (from 1981 to 1993) 23 publications, 15 of which were published in peer‐reviewed journals where metadata could be extracted. Yearly accumulation in publications returned by the *full document* search showed an exponential increase (Figure 2a), although the proportion of keystone predation papers compared to all ecological publications remained relatively constant through time (Figure 2a inset). By comparison, yearly accumulation in publications returned from the *title, abstract and keywords* in Scopus and the pre‐1993 Google Scholar searches showed a slower increase (Figure 2a) and a decline when compared to all published ecological literature (Figure 2a inset). This highlights that the proportion of ecological studies that only referred to keystone predation was constant, whereas the proportion of studies where keystone predation was of primary focus decreased over time. None of the 100 randomly selected *full document* Scopus publications provided empirical test data about keystone predation. Six of the 100 mentioned keystone predation in the main text body, where two papers were reviews, two described mathematical models, and two included empirical data (but not related to keystone predation tests). Furthermore, the remaining 94 publications were only identified in the search because they cited papers with ‘keystone predation’ in their titles. The cited keystone predation papers included Harley (2011, cited 33 times), Navarrete and Menge (1996, 25 times), Noonburg and Abrams (2005, 3 times), Leibold (1996), Sanford (2002), Amarasekare (2008), Calcagno et al. (2011), Zimmer et al. (2017) and/or Pang (2018, all one citation).

We therefore narrowed our review of keystone predation literature to publications returned from the third, more specific Scopus search (i.e., 58 papers with keystone predation in the title, abstract, and keywords) supplemented by the final Google Scholar older papers (another 15 papers, Figure 2b, green and orange bars). Of these 73 publications, 12.3% focused on theoretical modelling, 19.2% on reviews, books or book chapters, 17.8% were mensurative experiments (i.e., testing for keystone predation from observational data), and 50.7% experimentally manipulated predator abundances (Figure 2c, *χ* ^*2*^ = 26.45, *p* = 0.0005). Most of the manipulative experiments were done in situ (48.6%), followed by mesocosm (27.0%), laboratory experiments (18.9%) and a few studies combined field and laboratory experiments (5.4%, Figure 2d, *χ* ^*2*^ = 39.53, *p* = 0.0005). We also found that keystone predation was tested significantly more in freshwater (48%) and marine (44%) realms than in terrestrial realms (8%, *χ* ^*2*^ = 14.56, *p* = 0.001).

**FIGURE 3:**
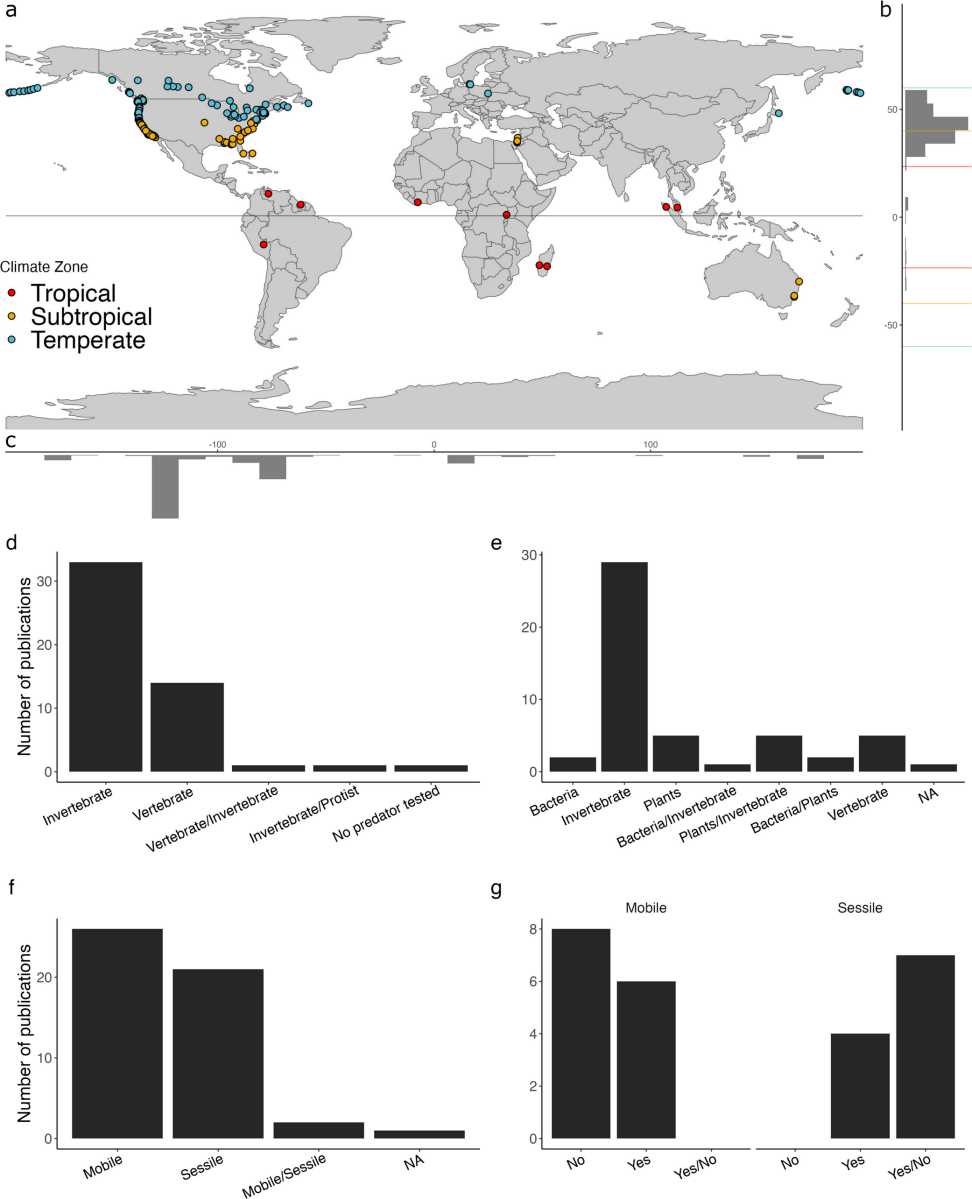
Keystone predation experiments. (a) Spatial distribution of keystone predation study sites (*n* = 333) extracted from 32 manipulative and 13 mensurative experimental publications (red = tropical, yellow = subtropical, blue = temperate latitude zones), sorted across (b) latitude and (c) longitude. (d) The number of publications that experimentally tested (manipulation or mensurative, *N* = 50) keystone predation grouped by type of predators, (e) type of prey competitors, and (f) prey mobility. (g) The number of publications where keystone predation was a main study objective (*N* = 25) and whether keystone predation was observed, grouped by the mobility of prey competitors.

Of the 50 studies that included mensurative or manipulative mesocosm or field experiments, 45 of them listed 335 georeferenced study locations. This spatial data showed that keystone predation has been tested on all continents except Antarctica, although the vast majority has been from North America (87.4%, Figure 3a–c) and temperate (59.4%) and subtropical (37.9%) climate zones (and only 2.7% from the tropics, Figure 3a–c, *χ* ^2^ = 165.26, *p* = 0.0005). The 50 studies included many different mobile predators, where invertebrates were studied more than vertebrates (68% vs. 28%, Figure 3d, *χ* ^2^ = 78.8, *p* = 0.0005). More prey communities were composed of invertebrates (58%) compared to plants (10%), vertebrates (10%) or combined invertebrate and plant communities (10%, Figure 3e, *χ* ^2^ = 83.1, *p* = 0.0005). Prey communities were relatively equally represented by mobile and sessile taxa independently (52% and 42%, respectively), whereas only 4% included both mobile and sessile prey together (Figure 3f, *χ* ^2^ = 16.13, *p* = 0.0005).

Of the 50 experimental studies, 37 manipulated the abundances of predators to test for causal and mechanistic links to keystone predation (Table 1). However, only 25 of the 37 studies tested for keystone predation as the primary research objective (Table 1), and of the remaining 12 studies, only three suggested, after data analyses, that keystone predation could partially explain the results. Furthermore, of the 25 manipulative experiments that explicitly tested for keystone predation, 8, 7, and 10 reported no support, context‐dependent support (i.e., varying with environmental conditions) or widespread support for keystone predation, respectively (Table 1). Of the 10 manipulative experimental studies that found widespread support for keystone predation, seven were from freshwater, two from terrestrial and one from a marine ecosystem. Furthermore, seven of these 10 studies manipulated abundances of invertebrate predators and reported impacts on invertebrate (2), plant (2), bacteria (2) or plant/invertebrate communities (1), whereas the remaining studies manipulated vertebrate predators and reported impacts on invertebrate (1), plants (1) or vertebrate prey communities (1). Widespread support for keystone predation was found on six mobile and four sessile communities (Figure 3g, Table 1). All eight unsupportive experiments tested for keystone predation on mobile communities (Table 1). Lastly, the seven studies that reported context dependencies were all done in marine systems, manipulating invertebrate predators and testing for impacts on sessile invertebrates (5) or sessile invertebrate/plant prey communities (2). In these studies, the context‐dependent keystone predation effects were related to variability in temperature (2 studies), wave exposure (2) or nutrient availability (3) processes, which impacted the productivity of the prey communities and the energy transfer between trophic levels.

**TABLE 1:** List of 37 experimental studies that manipulated predator presence to test keystone predation (KP), identified in Google Scholar and Scopus searches, including authors, publication year, study type, realm, predator taxa, community taxa (when available, s = strong competitor, w = weak competitor), community mobility, whether KP was the main study objective, and whether the study found support for KP.

Search engine	Authors	Publication year	Study type	Realm	Predator taxa	Community taxa	Community mobility	KP the main objective?	KP reported?
Google Scholar	Thorp and Bergey	1981	Field	Freshwater	Vertebrate (fish, turtles)	Invertebrate (benthic microinvertebrates)	Mobile	Yes	No
Google Scholar	Fairweather	1987	Field	Marine	Invertebrate (muricid whelk)	Invertebrate (s: snail, barnacle, limpet w: algae)	Mobile/sessile	No	No
Google Scholar	Strohmeier et al.	1989	Field	Freshwater	Vertebrate (spotted newt)	Invertebrate (benthic microinvertebrates)	Mobile	No	Proposed post hoc
Google Scholar	Blaustein and Marglit	1991	Laboratory	Freshwater	Invertebrate (fairy shrimp, ostrocods)	Bacteria/invertebrates (s: *Bacillus* sp. w: mosquito larvae)	Mobile	No	No
Google Scholar	Flecker	1992	Field	Freshwater	Vertebrate (characid fish)	Invertebrate (stream invertebrates)	Mobile	No	No
Google Scholar	Werner	1992	Laboratory	Freshwater	Vertebrate (wood frogs)	Not available	Not available	No	No
Google Scholar	Robles and Robb	1993	Field	Marine	Vertebrate/invertebrate (lobster, fish, whelk)	Invertebrate/plants (s: mussels w: epiphytes, coralline algae)	Sessile	Yes	Yes/no^a^
Google Scholar	Walters and Moriarty	1993	Field	Marine	Invertebrate/protists (nematodes, copepods, protists)	Bacteria/plants (benthic microbacteria, phytoplankton)	Mobile	No	No
Google Scholar	Wissinger and McGrady	1993	Field/laboratory	Freshwater	Invertebrate (dragonflies)	Invertebrate (damselfly)	Mobile	No	No
Scopus	Navarrete and Menge	1996	Field	Marine	Invertebrate (sea star, whelk)	Invertebrate (mussels)	Sessile	Yes	Yes/no^a^
Scopus	Robles	1997	Field	Marine	Invertebrate (lobster)	Invertebrate/plants (s: mussels w: algae)	Sessile	Yes	Yes/no^b^
Scopus	Sanford	1999	Field	Marine	Invertebrate (sea star)	Invertebrate (mussels)	Sessile	Yes	Yes/no^c^
Scopus	Steiner	2001	Mesocosm	Freshwater	Invertebrate (zooplankton)	Plants (phytoplankton)	Mobile	Yes	Yes
Scopus	Chalcraft and Resetarits Jr.	2003	Mesocosm	Freshwater	Vertebrate (fish, salamanders)	Vertebrate (frog and toad larvae)	Mobile	Yes	Yes
Scopus	Menge et al.	2004	Field	Marine	Invertebrate (sea star)	Invertebrate (mussels)	Sessile	Yes	Yes/no^b^
Scopus	Sarnelle	2005	Field	Freshwater	Invertebrate (zooplankton)	Plants (phytoplankton)	Mobile	Yes	Yes
Scopus	Jiang and Adams Krumins	2006	Laboratory	Freshwater	Invertebrate (Colpidium protist)	Bacteria (s: *Serratia* sp. w: *Bacillus* spp., unidentified sp.)	Mobile	Yes	Yes
Scopus	Schmitz	2006	Field	Terrestrial	Invertebrate (insect herbivores)	Plants (s: herb *Solidago* sp. w: grass *Poa* sp., other herb sp.)	Sessile	Yes	Yes
Scopus	Stevens and Steiner	2006	Mesocosm	Freshwater	Invertebrate (Colpidium protist)	Bacteria/plants (s: *Serratia* sp. w: *Closterium* sp.)	Mobile	Yes	No
Scopus	Declerck et al.	2007	Mesocosm	Freshwater	Invertebrate (substrate and seston zooplankton)	Plants (phytoplankton)	Mobile	No	Proposed post hoc
Scopus	Van Der Stap et al.	2008	Field	Freshwater	Invertebrate (carnivorous zooplankton)	Invertebrate (herbivorous zooplankton)	Mobile	Yes	No
Scopus	Eitam and Blaustein	2010	Mesocosm	Freshwater	Invertebrate (insect *Notonecta* sp.)	Invertebrate (s: *Daphnia* sp. w: *Moina* sp., *Ceriodaphnia* spp.)	Mobile	Yes	Yes
Scopus	Harley	2011	Field	Marine	Invertebrate (sea star)	Invertebrate (s: mussels w: barnacles, algae)	Sessile	Yes	Yes/no^c^
Scopus	Siepielski et al.	2011	Field	Freshwater	Vertebrate (fish, newts)	Invertebrate (damselflies)	Mobile	No	Proposed post hoc
Scopus	Freed et al.	2014	Laboratory	Freshwater	Invertebrate (Toxorhynchites mosquito)	Invertebrate (s: *Aedes japonicus japonicus* mosquito w: Aedes triseriatus mosquito)	Mobile	Yes	No
Scopus	Freed and Leisnham	2014	Laboratory	Freshwater	Invertebrate (Toxorhynchites mosquito)	Invertebrate (s: Aedes albopictus mosquito w: *Aedes japonicus japonicus* mosquito)	Mobile	Yes	No
Scopus	Rashidul Alam and Noda	2016	Field	Marine	Invertebrate (whelk)	Invertebrate/plants (s: *Balanas* sp. barnacle w: *Chthalmalus* sp. barnacles and algae)	Sessile	Yes	Yes
Scopus	Zimmer et al.	2016	Laboratory	Marine	Invertebrate (sea star)	Invertebrate (mussels)	Sessile	No	No
Scopus	Zimmer et al.	2017	Field/laboratory	Marine	Invertebrate (sea star)	Invertebrate (mussels)	Sessile	No	No
Scopus	Canter et al.	2018	Laboratory	Freshwater	Invertebrate (protozoans)	Bacteria (pitcher plant inquiline bacteria)	Mobile	Yes	Yes
Scopus	Davidson and Dorn	2018	Mesocosm	Freshwater	Invertebrate (crayfish)	Invertebrate (s: *Pomacea maculata* snail w: *Pomacea paludosa* snail)	Mobile	Yes	No
Scopus	Pringle et al.	2019	Mesocosm	Terrestrial	Vertebrate (curly tailed lizards)	Vertebrate (s: green anole w: brown anole)	Mobile	Yes	No
Scopus	Nieoczym et al.	2020	Mesocosm	Freshwater	Vertebrate (cyprinid fish)	Invertebrate (chironomid benthic macroinvertebrates)	Mobile	Yes	Yes
Scopus	Sullivan‐Stack and Menge	2020	Field	Marine	Invertebrate (sea star)	Invertebrate (mussels)	Sessile	No	No
Scopus	Menge et al.	2021	Field	Marine	Invertebrate (sea star)	Invertebrate (s: mussels, w: algae)	Sessile	Yes	Yes/no^b^
Scopus	Stemp et al.	2021	Mesocosm	Freshwater	Vertebrate (salamander)	Vertebrate (frog and toad larvae)	Mobile	Yes	No
Scopus	Ratajczak et al.	2022	Mesocosm	Terrestrial	Vertebrate (bison)	Plants (grasses, forbs)	Sessile	Yes	Yes

*Note:* Certain studies observed that keystone predation was affected over gradients of (a) wave action, (b) nutrients and energy transfer between trophic levels, or (c) temperature, signalling context dependence.

## 4 Discussion

**FIGURE 4:**
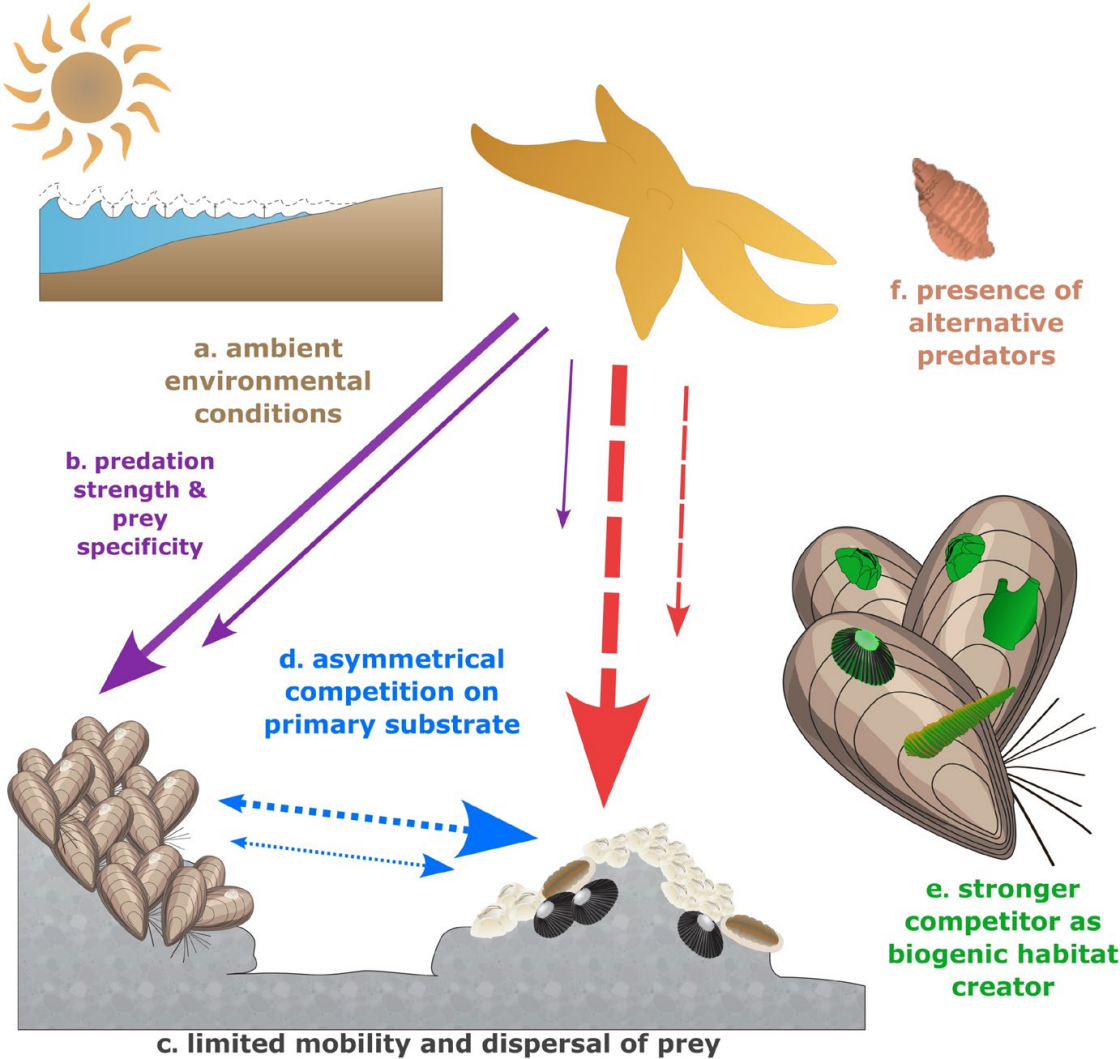
Context dependencies in keystone predation. The strength of keystone predation can depend on (a) the ambient abiotic environmental conditions, (b) the strength of predation on the superior prey competitor (and possible prey switching), (c) the mobility and dispersal of prey (weak vs. strong) (d) the degree of asymmetrical competition between prey species (weak vs. strong), (e) whether the competing prey species build complex secondary (biogenic) substrates to control biodiversity through habitat formation and (f) the presence of alternative predators and their prey preference. Arrows depict the direction of impact between levels, rather than the flow of energy. Species graphics are from Integration and Application Network (ian.umces.edu/media‐library). They are licensed under Attribution‐ShareAlike 4.0 International (CC BY‐SA 4.0).

Keystone predation is often cited as a critical process that shapes ecological communities in the primary literature (see our results section), textbooks (Krebs 2009; Mittelbach and McGill 2019; Smith 2015), and in reviews of indirect species effects (Menge 1995; Paine 1995; Wootton 1994). However, despite these widespread references and citations, we found limited empirical evidence in the few studies that have explicitly tested for keystone predation. For example, only 37 publications manipulated predator abundances and discussed the results in the specific context of keystone predation; only 25 of these studies did so as the primary study objective, and only 10 of these studies found consistent support for keystone predation. Perhaps our most central finding is therefore the lack of well‐defined empirical tests of keystone predation, particularly outside North America. The lack of empirical research limited our ability to identify generalities or gauge the importance of keystone predation across realms and environmental conditions. Nonetheless, the few identified empirical studies demonstrated that keystone predation has been found across realms, bioregions and latitudes (Rashidul Alam and Noda 2016; Ratajczak et al. 2022; Schmitz 2006), depends on the characteristics of the predator and prey communities (Canter et al. 2018; Chalcraft and Resetarits 2003; Eitam and Blaustein 2010; Steiner 2001), and varies in importance depending on local environmental conditions (Harley 2011; Menge et al. 2021; Sanford 1999). Below, we discuss our findings from the identified empirical and theoretical/modelling literature to better understand when, where and how keystone predation may modify or control ecological community structure (Figure 4).

### 4.1 Environmental and Ecological Context

Environmental conditions have long been suggested to modulate keystone predation (Figure 4a; Harley 2011; Leibold 1996; Sanford 1999). For example, high nutrient levels increased growth and predation resistance for the strong apple snail competitor, which altered predation pressures and weakened keystone predation (Davidson and Dorn 2018). By contrast, the high productivity of competitors, which typically increases with nutrient availability, increased predation rates of the sea star *Pisaster* on mussels in rocky intertidal communities and thereby increased keystone predation (Menge et al. 2021). Alternatively, *Pisaster* predation can also decrease under high temperatures and thereby reduce keystone predation (Figure 4b; Sanford 1999) with cascading impacts on communities of intertidal sessile seaweeds and invertebrates (Harley 2011). These examples suggest that the strength of keystone predation varies in space and time, as ambient environmental conditions experience cyclic or stochastic variation (Noonburg and Abrams 2005). By contrast, Ratajczak et al. (2022) showed, in a 30‐year study, that bison ( *Bison bison* ) consistently increased species richness of grasses through keystone herbivory, despite variation in rainfall, including a 2‐year drought. The long‐term persistence of competing prey species was attributed to niche partitioning between grass and forbs, suggesting species traits can also modulate keystone predation (Menge et al. 2021).

### 4.2 Species Traits

Several studies have suggested that species traits affect keystone predation (Leibold 1996; Menge et al. 1994, 2021). Our review highlighted that keystone predation predominantly has been observed in prey communities with limited mobility, including sessile species (Menge 1995; Menge et al. 2021; Navarrete and Menge 1996; Rashidul Alam and Noda 2016), plankton (Sarnelle 2005), larvae (Chalcraft and Resetarits 2003) or in confined areas for mobile prey, such as within pitcher plants (Table 1; Canter et al. 2018). The limited mobility of prey species (Figure 4c) is likely to increase the importance of allocating resources to predator deterrence over fast growth, a fundamental trade‐off in the broader understanding of competition dynamics (Figure 4d; Leibold 1996; Van Der Stap et al. 2008; Vance 1978). Indeed, several studies have suggested that the trade‐offs between predator defences vs. prey productivity modify outcomes from keystone predation (Menge et al. 1994, 2021). For example, as mentioned above, bison facilitate coexistence between grasses and forbs (also during the two‐year drought) partly through resource partitioning of soil water extraction (Ratajczak et al. 2022). By contrast, in the absence of bison, grasses with shallow, denser roots outcompeted forbs for limited water during dry periods (Ratajczak et al. 2022). Furthermore, facilitation processes within and between prey and other community members can also modify the strength of keystone predation. For example, species richness in rocky intertidal communities can increase (instead of decrease) after the removal of *Pisaster* sea stars and subsequent mussel space pre‐emption, because mussel create complex biogenic habitat (i.e., secondary substrate) that facilitates many sessile epibionts and mobile invertebrates (Harley 2011; Lafferty and Suchanek 2016; Lohse 1993). Behavioural traits or species plasticity to changing environmental conditions may also modify keystone predation. For example, Pringle et al. (2019) reported that, in the presence of predators, fear‐induced avoidance altered competition dynamics between lizard prey, contributing to the absence of keystone predation. Stemp et al. (2021) suggested that geographic variability in predator traits, such as predation rates and aggression, led to different salamander predation pressures and selectivity, contributing to the absence of keystone predation in their salamander‐frog larvae communities. In short, traits of both predators and their prey can modify the importance of keystone predation, although targeted experiments are needed to understand whether these findings can be generalised into predictive models.

### 4.3 Connectivity and Dispersal

While mobility can influence species susceptibility to keystone predation, dispersal and connectivity among patches can also regulate communities (Haegeman and Loreau 2014; Tonkin et al. 2018), including by altering species interactions (Mohd et al. 2017; Strecker and Arnott 2010; Wieters et al. 2008), and therefore keystone predation. However, it is difficult to quantify and test for dispersal and connectivity in field experiments (Allan et al. 2013; Bernard et al. 2021; Thomas et al. 2011), and linkages to keystone predation have so far mainly been addressed in simulations (Amarasekare 2008; Shurin and Allen 2001). For example, Amarasekare (2008) modelled that coexistence between prey species was more likely to be controlled by keystone predation when inferior competitors could not disperse. This finding is supported by empirical studies that have shown keystone predation is stronger in communities with sessile prey in rocky intertidal systems (Menge 1995; Menge et al. 2021; Navarrete and Menge 1996; Rashidul Alam and Noda 2016), grasslands (Ratajczak et al. 2022; Schmitz 2003) and in pitcher plant inquiline communities (Canter et al. 2018). Dispersal limitations in otherwise mobile prey could perhaps also explain that keystone predation has been documented in microscopic communities, such as bacteria (Jiang and Adams Krumins 2006), microscopic invertebrates (Eitam and Blaustein 2010; Nieoczym et al. 2020; Sarnelle 2005) and frog larvae (Chalcraft and Resetarits 2003). Although logistically challenging, we suggest that incorporating dispersal and connectivity of prey species in future tests of keystone predation could provide key insights into understanding ecological context dependencies (Amarasekare 2010; Haegeman and Loreau 2014; Leibold et al. 2004; Thompson et al. 2020).

### 4.4 Confusions About Terminology

Here, we reviewed keystone predation as the process whereby consumers preferentially eat strong competitors to indirectly facilitate weaker competitors and increase the coexistence of prey competitors (see Box 1). However, we also found examples of terminological confusion. First, the term keystone ‘predation’ suggests that the consumer is always a carnivore, but several studies address the effects of herbivores on plant communities (Poelman and Kessler 2016; Rashidul Alam and Noda 2016; Ratajczak et al. 2022; Schmitz 2006). Indeed, keystone ‘herbivory’ is likely to be widespread and ecologically important, as suggested by Thomsen (2022). In their proof‐of‐concept meta‐analysis, the author showed aquatic herbivores indirectly facilitate seaweeds and corals by preferentially grazing on faster‐growing, competitively superior weeds and epiphytes. Similarly, parasites can preferentially infect dominant competitors (keystone parasitism), although their effects, compared to predation, will likely be slower, less lethal and the cascading community impacts, therefore, more subtle (Fenton and Brockhurst 2008; Grewell 2008). To avoid this ambiguity, it has been suggested to broaden keystone predation to keystone consumption and reserve the more precise terminology (keystone predation/herbivory/parasitism) for specific case studies (Fenton and Brockhurst 2008; Grewell 2008; Ratajczak et al. 2022; Thomsen 2022; Thomsen et al. 2010).

Second, keystone consumption can both increase and decrease species richness depending on how the prey community and associated species are sampled, which may be biased by a researcher's background and perspective (Levin et al. 2021). For example, some competing prey species provide secondary (biogenic) substrates to facilitate species that would otherwise be outcompeted on primary (abiotic) substrates and, ultimately, weaken keystone predation (Figure 4e.; Harley 2011; Lafferty and Suchanek 2016; Lohse 1993). More specifically, richness has been reported to both increase under keystone predation (e.g., when mussel‐associated plants and animals are counted) or decrease, such as when effects are only measured on competitors on primary substrates, as discussed in detail for Paine's original study (Lafferty and Suchanek 2016; Levin et al. 2021; Lohse 1993; Suchanek 1985). It is therefore crucial for researchers to clearly state their precise hypotheses about keystone predation and what type of prey community (and associated organisms) they measure, removing potential misinterpretations (Levin et al. 2021).

Third, terminological confusions can be exaggerated by limited knowledge of species interactions that lead to misidentification of keystone predation. For example, keystone predation and apparent competition can only be differentiated by measuring energy flow between trophic levels (compare arrows in Box 1 Figure 1a vs. Figure 1c). Recognising and separating keystone predation and apparent competition, therefore, requires targeted experiments related to predator functional responses, alternative predator impacts (Figure 4f; Fauth and Resetarits 1991; Navarrete and Menge 1996), prey selectivity and switching and competitive interaction strengths (Bogdziewicz et al. 2020; Campbell et al. 2009; Coblentz and DeLong 2020; Underwood 2000).

Finally, as described in the introduction, ‘keystone predation’ can be confused with ‘keystone species’, where the latter refers broadly to *any* species, irrespective of trophic position, that has a disproportionate impact on other species relative to its abundance (Bond 1994; Mouquet et al. 2013; Power et al. 1996). The broader keystone species concept can, therefore, in addition to being a predator, include mutualists, facilitators and competitors involved in myriads of interactions like keystone predation, keystone mutualism, trophic cascades, facilitation cascades or competition cascades (Thomsen et al. 2010). The keystone species concept is much more widespread than keystone predation (70,800 vs. 1910 hits on Google Scholar, November 5, 2023) and has been dissected and reviewed many times (Cottee‐Jones and Whittaker 2012; Libralato et al. 2006; Mills and Doak 1993; Paine 1995; Power et al. 1996). Some papers testing for keystone species also address keystone predation, even if these papers do not use the term keystone predation or describe the process explicitly (Box 1). For example, Fauth and Resetarits (1991), Morin (1981, 1983), and Wilbur et al. (1983) identified keystone predation in isolated ephemeral ponds where salamanders and newts consumed strong competitor anuran larvae, which facilitated weaker competitors. Similarly, the crown‐of‐thorns sea star (*Acanthaster cf. solaris*) has been described as a keystone species that preferentially consumes strong coral competitors like *Acropora* and *Montipora* and thereby indirectly facilitates inferior *Porites* corals (Pratchett et al. 2017). However, these papers did not use keystone predation terminology and were therefore not identified in our literature searches or included in our data analyses. Furthermore, none of the grazer‐seaweed papers identified in Thomsen (2022) used keystone predation, even though they reported indirect facilitations of weak competitors in the presence of grazers that preferentially consumed the stronger competitors. These examples highlight that future global meta‐analyses of keystone predation should combine wider search criteria (e.g., keystone species/grazer/predator) with expert knowledge in systems with known strong specialist consumers.

### 4.5 Alternative Analytical Methods

Traditional methods for testing keystone predation have focused on manipulating the abundances of potential keystone predators/consumers. However, as discussed, environmental conditions, species traits and dispersal can modify the strength of keystone predation. Incorporating broad environmental gradients and connectivity in manipulative experiments is, unfortunately, extremely challenging. Statistical tools, like path analysis, network theory (Bascompte and Jordano 2007; Windsor et al. 2023), and joint species distribution models (Pollock et al. 2014), may provide supplementary analytical methods to quantify the strength of keystone predation and potential context dependencies, from pre‐existing large biological databases. For example, multilayer networks may reveal keystone predation mechanisms by modelling interaction strengths between prey at one level, which may respond to trophic interactions by a species at another level (Dehling 2024; Kéfi et al. 2015; Timóteo et al. 2022). Combining multilayered network analysis with conventional species‐level network metrics can then reveal the importance of certain species (Hutchinson et al. 2019; Pilosof et al. 2017). For example, betweenness centrality quantifies which species connect the rest of the network, highlighting their crucial role in maintaining community structure (Genrich et al. 2017; Mello et al. 2015). Combining multilayer centrality with network sensitivity analysis (Jordán et al. 2006), which quantifies network change through the systematic removal of species, may help provide detailed insights into keystone predation's strength, the species included in it and potential cascading impacts on the wider community. Similarly, joint species distribution models can use community composition and species abundances, environmental covariates, species traits, and spatial data structures to predict community dynamics and model latent species interactions, and thereby identify keystone predation and context dependencies (Pollock et al. 2014; Tikhonov et al. 2017, 2020). Finally, these analytical methods are also useful for analysing keystoneness as a continuous trait (McGill and Brown 2007), although such analyses are more relevant for future reviews of the keystone species concept (rather than the keystone predation process). In short, traditional manipulative and mensurative experiments, when combined with new statistical tools, provide better pathways to identify, quantify and predict keystone predation.

### 4.6 Future Studies

An important goal of our review was to identify research gaps to be addressed in future studies that enhance our understanding of keystone predation. Beyond our suggestions for advanced analytical methods, we suggest five areas of future research effort. First and foremost, more experiments should aim to manipulate the abundance of a single predator species (rather than a multispecies guild of predators) and measure the impact on well‐defined communities of potential prey species (Canter et al. 2018; Menge et al. 2021; Ratajczak et al. 2022). Where possible, these experiments should be combined with manipulative experiments that quantify predator–prey preferences and competitive hierarchies between potential prey (Carmona et al. 2019; Connell 1961; Geange et al. 2013; Wray et al. 2021). Second, future studies should target understudied regions, systems, habitats, climates and taxa identified here (see Figures 2 and 3) to broaden our understanding of keystone predation across space and time (e.g., testing the effects of vertebrate consumers on vertebrate prey in tropical climates on the African continent). Third, experiments should test for context dependencies that we have identified. These experiments could involve factorial approaches that cross predator manipulation with other manipulated test factors (like fertilisation) or repeat experiments along environmental stress gradients to identify how important processes are under different conditions (Clemente and Thomsen 2025; Davidson and Dorn 2018). Fourth, we see a great opportunity to build a comprehensive database of papers that also qualify as tests of keystone predation but were not captured in our literature search. We provide a few examples (e.g., Fauth and Resetarits 1991; Morin 1981; Pratchett et al. 2017; Wilbur et al. 1983), but a more systematic and exhaustive effort is required, including using diffusive habitat‐specific expert knowledge (i.e., global coalitions or working groups of ecologists spanning aquatic and terrestrial habitats). Building this knowledge would provide a solid foundation for future meta‐analyses, as done for other indirect interactions, like trophic cascades or facilitation cascades (Shurin et al. 2002; Thomsen et al. 2018). Fifth, with an advanced understanding of keystone predation, new studies can apply keystone predation processes to management and conservation efforts, like those successfully done for herbivory (Xu et al. 2023), trophic cascade theory in managing marine protected areas (Kumagai et al. 2024) or coplanting of foundation species to improve restoration of ecosystem function (Zhang et al., 2025).

## 5 Conclusion

Keystone predation has been widely cited as a key ecological mechanism controlling community structures (Menge et al. 1994, 2021; Mittelbach and McGill 2019; Wootton 1994), but is supported by limited theoretical (Amarasekare 2008; Leibold 1996; Noonburg and Abrams 2005) and experimental studies (Canter et al. 2018; Ratajczak et al. 2022; Schmitz 2006; Steiner 2001). Still, we showed that keystone predation has been documented across realms and appears to be most prevalent in sessile and dispersal‐limited communities (Table 1, Figure 3f). However, we also conclude that only a few studies have explicitly tested for keystone predation thereby limiting our ability to identify generalisations (Lawton 1999; Menge et al. 2021; Terborgh 2015). To address these gaps, we have identified several areas of promising future research, including new manipulative experiments, filling geographic gaps and advanced analytical methods. An improved understanding of keystone predation may ultimately enable better targeted management in response to the growing biodiversity crisis (Harley 2011; Jan et al. 2023; Worm and Lotze 2021).

## Author Contributions

**Anthony J. Gillis:** conceptualization (equal), data curation (lead), formal analysis (lead), investigation (lead), methodology (equal), visualization (equal), writing – original draft (equal). **Mads S. Thomsen:** conceptualization (equal), data curation (supporting), formal analysis (supporting), funding acquisition (equal), investigation (supporting), methodology (equal), supervision (equal), visualization (equal), writing – review and editing (equal). **Jonathan D. Tonkin:** conceptualization (equal), data curation (supporting), formal analysis (supporting), funding acquisition (equal), investigation (supporting), methodology (equal), supervision (equal), visualization (equal), writing – review and editing (equal).

## Conflicts of Interest

The authors declare no conflicts of interest.

## Supporting information


**Data S1:** Subset of randomly selected publications returned by our full document SCOPUS search, which was used to assess the testing and discussion of keystone predation in ecological literature. This file contains publication year, author list, publication title, journal name, and section where keystone predation was mentioned (i.e., title, abstract, keywords, and main text and/or citation), and the keystone predation‐titled publications cited within each.

**Data S2:** Full list of keystone predation publications returned from our specific “title, abstract, & keywords” SCOPUS search, as well as the Google pre‐1993 search, which was used to assess whether empirical evidence supports keystone predation. This file contains the search database where the publication was found, publication year, authors' list, article title, journal name, relevant experimental metadata, such as experiment type, location, realm community/predator taxa, and mobility. The file also contains whether the publication authors say keystone predation was observed in their experiments or not.

**Data S3:** Coordinates for the individual study sites (*n* = 335) from the 45 studies returned from the “title, abstract, & keywords” SCOPUS search, as well as the Google pre‐1993 search, which was used to better understand the global distribution of keystone predation studies. This file contains a relational publication identifier (to the keystone predation‐specific publications, see ST2KPPubs file), the study's publication year, lead author, coordinates in decimal degrees and a publication‐provided site name.

## Data Availability

The data that supports the findings of this study is available in the Supporting Information of this article.
